# Supplementary material for: The Microbiota of the Outer Gut Mucus Layer of the Migrating Northeast Arctic Cod (Gadus morhua) as Determined by Shotgun DNA Sequencing
Source: Microorganisms. 2024 Oct 31;12(11):2204. doi: 10.3390/microorganisms12112204 (PMC11596785; doi:10.3390/microorganisms12112204)
Supplement: Supplementary file 1 [file microorganisms-12-02204-s001.zip › microorganisms-3250715-supplementary.pdf]

Supplementary Materials

The microbiota of the outer gut mucus layer of the migrating Northeast Arctic cod (*Gadus morhua*) as determined by shotgun DNA sequencing

Text S1: Method for DNA extraction of intestinal mucus from six Atlantic cod:

Total DNA was isolated from the collected mucus sample of six migrating Northeast Atlantic cod. DNA isolation proved to be very challenging, typically producing very small amounts of DNA of poor quality. Thus, three DNA isolation protocols (Fig. 5) were tested and modified before DNA of sufficient quantity and quality was obtained. In the first attempt, the DNazol method was used, followed by extraction with phenol:chloroform:isoamyl alcohol (25:24:1 saturated with 10 mM Tris, pH 8.0, 1 mM EDTA; Sigma-Aldrich) followed by precipitation with 2.5 vol 96% ethanol (stored at –20 °C) and 0.1 vol 3M Sodium Acetate, pH 5.2. The DNazol method protocol was optimized by incorporating additional steps. A lysozyme treatment was introduced by adding 200 µL of 100 mg/mL lysozyme to the samples, followed by incubation for 10 minutes at 37 °C. After the elution step, the samples were treated with 5 µL of RNase A (5 µg/µL) and incubated for 30 minutes at 37 °C to ensure the degradation of RNA. Typically, this resulted in DNA concentrations ranging from 7–17.6 ng/µL with acceptable OD260/280 (~1.8) and OD 260/230 (2.0–2.2) ratios. However, the samples did not yield clear DNA bands on agarose gels and were not successful when subjected to Illumina sequencing using Swift Turbo library preparation. It is possible that the presence of RNA in the samples led to an overestimation of the DNA concentration despite RNase A digestion. We then tried to extract mucosal DNA using the High Pure PCR Template Preparation Kit (Roche, Basel, Switzerland), but this method also did not yield high-quality DNA. Finally, we used the FastDNA™ Spin Kit (MP Biomedicals) for extracting DNA from the mucus, in a similar manner as described earlier. Firstly, 200-250 mg of mucus were added per E-lysing matrix tube. Four tubes were used per fish for increasing the DNA yield. The samples underwent homogenization using the FastPrep®-24 system (MP Biomedicals) for a duration of 10-15 seconds at a setting of speed 4. Instead of employing a vortex, we opted for a manual rotation of the samples to always mix them gently. For the re-suspension of the DNA pellet, we carefully employed a micropipette equipped with the largest available tips to reduce the risk of DNA shearing. We extended the duration of the final centrifugation step, which involved a wash with SEWS-M, from one minute to two minutes. The samples were eluted in 200 µl of clean distilled water. RNA was removed by incubating the eluted samples with 1 µL of RNase cocktail (500 U/ml RNase A and 20,000 U/ml RNase T1 g/mL) for 10 minutes at 37 °C. Secondly, DNA purification commenced with phenol-chloroform extraction where 200 µL of phenol-chloroform was added to an equal volume of DNA solution. Tubes were mixed by inversion, not vortexing, Centrifugation at room temperature for 5 minutes at 14,000 rpm allowed phase separation. A portion of the lower phase was discarded to minimize phenol contamination and maximize DNA yield. An additional centrifugation at the same conditions ensured clear phase distinction. The aqueous phase (upper layer), approximately 180 µL, was transferred to a new tube, avoiding phenol-chloroform uptake. DNA was then precipitated overnight with 1/10 volume of 3M sodium acetate (pH 5.2) and 2 volumes of cold 100% ethanol at -20°C, concentrating the DNA and eliminating contaminants. Samples were gently mixed by tube inversion. Next a centrifugation was performed at 14,000 rpm and 4 °C for 30 minutes, after which the supernatant was discarded. DNA pellets were washed with 500 µL of pre-chilled 70% ethanol and re-centrifuged for 5 minutes at 14,000 rpm and 4 °C. The supernatant was removed, and the tubes were quick spin to collect residual ethanol, which was then pipetted out. DNA pellets were dried using a Speed Vac for 3 to 7 minutes at room temperature. Finally, pellets were resuspended in 20 µL of 10 mM Tris buffer (pH 7.5) without EDTA. Yields from 0.29 to 10 ng/µL (averaging 1.77 ng/µL among the six samples) were obtained in a total volume of ≤ 20 µL, and with acceptable DNA quality. Table 3 shows DNA isolation outputs for six mucus samples that were subjected to Illumina MiSeq sequencing. These samples were DNA sequenced at the Norwegian Sequencing Centre (NSC) using the Illumina MiSeq platform. Due to the low yield of

the isolated total DNAs, sequencing was done with 250 bp (Smart ThruPlex for low-input samples) paired-end reads. At first by subjecting samples to Smart ThruPlex library preparation for low-input samples, and then by running the 250 bp paired-end sequencing using MiSeq Reagent v.2 (500 cycles). The purity of the samples was monitored by Nanodrop 2000c (Thermofisher Scientific, USA) and the final DNA concentration was determined with a Qubit 2.0 Fluorometer (ThermoFisher Scientific).

Supplementary Figure S1:

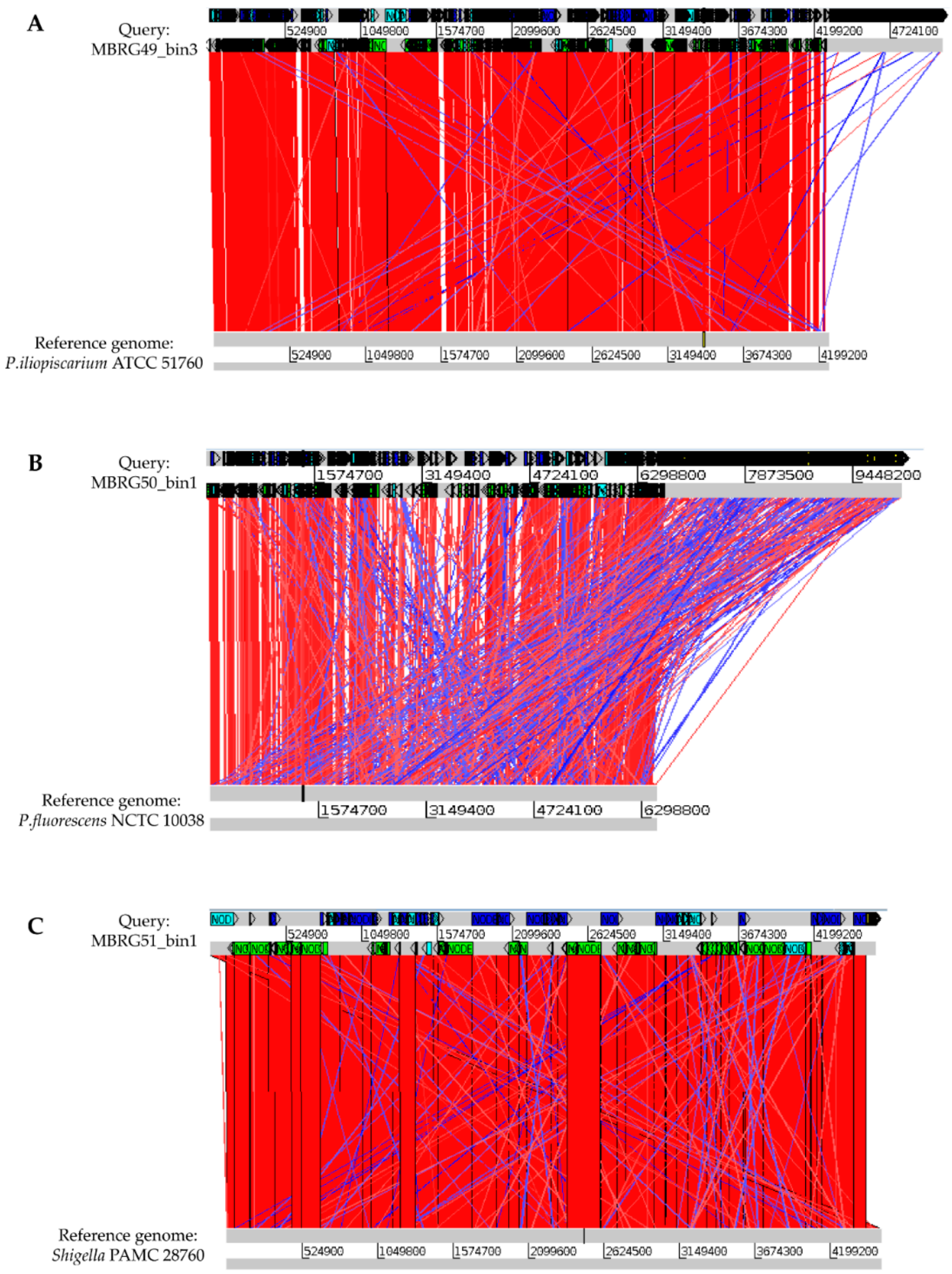

Figure S1. Visualization comparison of three Metagenome Assembled Genomes (MAGs) against reference genomes using ACT, the Artemis comparison tool. (A) MBRG49_bin3 (from Type II profile) comparison with *Photobaterium iliopiscarium* ATCC 51760, (B) MBRG50_bin1 comparison with *Pseudomonas fluorescens*, and (C) MBRG51_bin1 comparison with *Shigella* PAMC 28760. Note that the comparisons in A and B each contain a series of MAG contigs located to the far right of the sequence that are missing from, or do not match well with the corresponding reference genome. The comparison in C show that MBRG51_bin1 is highly similar to Shigella PAMC28760.
